# An unusual and life-threatening presentation of a large GIST

**DOI:** 10.1016/j.ijscr.2022.107666

**Published:** 2022-09-17

**Authors:** Nasrin Saeidi, Yousef AlAli, Reem Boushehry, Sarah Al Safi

**Affiliations:** Department of Surgery, Al-Adan Hospital, Kuwait

**Keywords:** GIST, gastrointestinal stromal tumour, GI, gastrointestinal, CTA, computed tomography angiography, TAE, transcatheter arterial embolisation, Case report, Gastrointestinal stromal tumours, Gastrointestinal bleeding, Second-look surgery, Jejunal tumour

## Abstract

**Introduction:**

Gastrointestinal stromal tumours (GIST's) are rare tumours of the alimentary tract. They are often discovered incidentally during imaging or intra-operatively. In rare instances, they present acutely with life threatening gastrointestinal (GI) bleeding requiring emergency surgical intervention.

**Case presentation:**

A 47-year-old gentleman, who is an ex-smoker with normal body mass index (BMI), presented with acute onset of epigastric pain, dizziness, and multiple episodes of melaena. The patient deteriorated rapidly and urgent endoscopy revealed active retrograde bleeding from beyond the duodenojejunal junction. Computed tomography angiography (CTA) suggested a highly vascular ileal exophytic mass resembling a GIST. Emergency exploratory laparotomy was conducted where hemostasis was achieved via segmental enterectomy of the mass that was unexpectedly jejunal in origin. During recovery, he encountered post-operative complications that were managed conservatively and eventually was discharged with a referral to the national cancer centre.

**Clinical discussion:**

The clinical presentation of GIST is based on its size and location. Definitive diagnosis of GIST relies on histopathological findings although the clinical presentation and imaging, in particular CTA, can aid in its diagnosis. Management of GIST differs depending on the clinical presentation, size, location and whether metastasis is present. Surgical resection is the standard of treatment; however, Imatinib could be used for non-resectable tumours as well as in cases of recurrence, metastasis or as an adjuvant chemotherapy.

**Conclusion:**

It is important to acknowledge that small GISTs are often asymptomatic while larger ones may present with non-specific symptoms which can be misleading. This could potentially delay the diagnosis and thus treatment of GIST which can be detrimental in acute cases as illustrated here. It is important to have GIST as one of the differentials when faced with a patient presenting with non-specific GI symptoms.

## Highlights

• Although an acute presentation of GIST is rare, it must be recognised early to prevent further morbidity and mortality.
• The overlap between the symptoms of GIST and other GI disorders could lead to an uncertain diagnosis and thus unfavourable outcome.
• Imaging modalities along with immunohistochemical studies have eased the diagnosis of GIST
• The characteristics of the tumor largely influence the treatment pathway.

## 1 Introduction

GISTs are rare tumours of the alimentary tract, which may occur anywhere along the digestive tract with higher occurrences in the stomach and small intestine [1], [2], [3]. It is important to note that the location of the tumour may contribute to the presenting symptoms [2], [4]. Nonetheless, it is often found incidentally during radiological imaging or intraoperatively [2], [4]. Historically, these tumours were classified as leiomyoma, schwannoma, or leiomyosarcoma [1], [5]. In the 1980s, they began to be recognised as a distinct group of mesenchymal tumours based on their unique immunohistochemical profile [5]. In 1998, Hirota et al. discovered that perhaps this unique profile was due to a gain-of-function mutation in the proto-oncogene receptor tyrosine kinase within the interstitial cells of Cajal [5]. In fact, up to 95 % of GIST tumours stain positive for KIT which is often negative in other mesenchymal tumours [1], [4]. These advances have significantly revolutionised the management of GIST through the introduction of molecular targeted therapy to the mainstay of treatment: surgical resection [2], [3], [4], [6]. Herein, we report a case of a 47-year-old male patient, who presented acutely with massive GI bleeding with an unexpected finding of a mass on CT angiography suspicious of a GIST tumour. This case report has been reported in line with the SCARE criteria [7].

## 2 Case presentation

A 47-year-old ex-smoker male with a normal body mass index (BMI) of 20, had peptic ulcer disease, treated with triple therapy in 2016. The patient presented to the emergency department with a 1-day history of severe epigastric pain, dizziness, and multiple episodes of melaena. On initial physical examination, the patient was hypotensive with blood pressure of 92/62 mmHg, pulse 60 bpm, temperature of 37 degrees Celsius, oxygen saturation of 100 % on room air. The initial examination was significant for a mildly distended abdomen with tenderness in the epigastric region and no signs of guarding or rigidity. A digital rectal examination confirmed the presence of melaena.

**Fig. 1:**
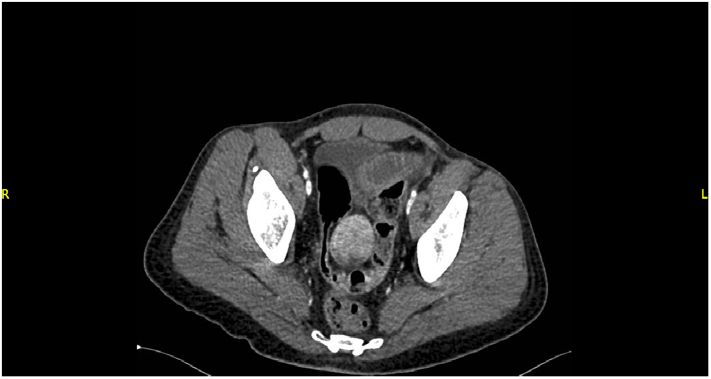
CT-angiogram showing a well-circumscribed, highly vascular mass (red arrow). (For interpretation of the references to colour in this figure legend, the reader is referred to the web version of this article.)

Initial laboratory investigations were as follows: Haemoglobin of 7.3 g/dL, pH 7.32, lactate 1.98 mmol/L, white cell count 13 × 10^9^/L, platelets 74 × 10^9^/L, international normalised ratio of 1.54. The liver function test and biochemistry results were unremarkable. The patient was resuscitated with intravenous fluid and packed red blood cells (PRBCs) in the emergency department. An urgent gastroenterology consultation was initiated in addition to intensive care unit (ICU) assessment. He was immediately started on omeprazole infusion. The patient remained hypotensive; noradrenaline infusion was started by the ICU team, and he was shifted to the ICU. An urgent esophagogastroduodenoscopy (EGD) was performed in the ICU which showed grade II distal esophagitis, gastric erythema, first part of duodenum ulcer with clean base and no stigmata of recent bleeding. However, there was retrograde fresh blood coming beyond the duodenojejunal junction. Despite continuous resuscitation, the haemoglobin dropped to 5.8g/dL, and the patient was hemodynamically unstable. An urgent abdominal CTA revealed a well circumscribed, highly vascular, distal ileum mass with an exophytic component (Fig. 1).

**Fig. 2:**
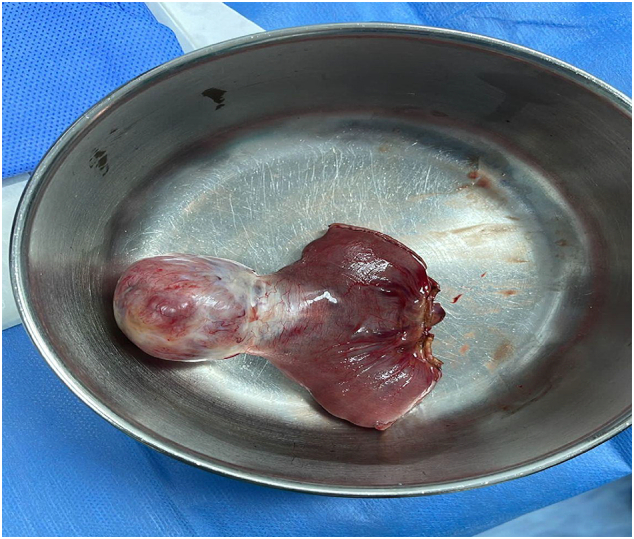
The resected tumour.

Upon reassessment, a new finding of generalised abdominal rigidity was discovered. Emergent exploratory laparotomy was performed via midline incision. The intraoperative findings revealed a jejunal exophytic mass about 4X3X3 cm and around 120 cm from the duodenojejunal junction with minimal serous fluid intraperitoneally (Fig. 2). Damage control was performed with only resection of a jejunal mass due to the hemodynamic instability of the patient, and presence of high dose of inotropic and vasopressor supports with the intent for a second look after resuscitation and stabilisation. The patient remained intubated and closely observed in the ICU. After 48 h, the inotrope had been discontinued, and the haemoglobin level had stabilised. He was taken to the operating theatre for a second look, during which 10 cm of small bowel loops were resected from jejunal edges along with the mesentery with side-to-side anastomosis, placement of Malecot drainage catheter, and closure of the abdomen. The patient was then observed in the ICU for 3 days, after which he was transferred to a general surgical ward in a stable condition. During his hospital stay, the patient developed a wound infection, which was managed with ceftriaxone (2 g IV) with twice-daily wound cleansing and dressing change. He also developed a partial bowel obstruction, secondary to an ileus. The obstruction was treated conservatively with a nasogastric tube and a trial of Gastrografin.

**Fig. 3:**
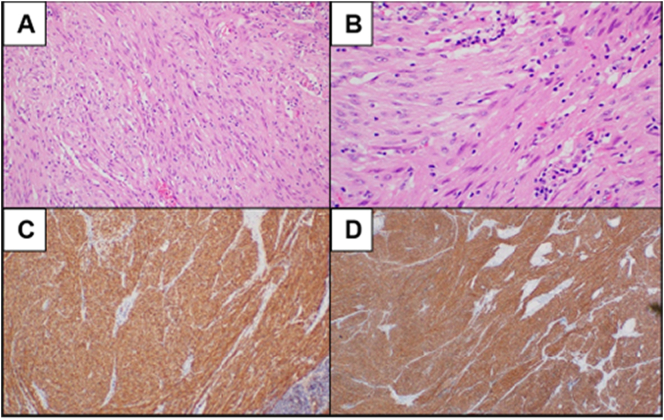
Histopathology findings. (A and B) Spindle and epithelioid cells, (C) positive CD117 staining, (D) positive DOG-1 staining.

The initial histopathology findings of the jejunal specimen were consistent with grade 1 GIST. Grossly, the specimen consisted of a brown segment of small bowel, measuring 7 cm in length and 3 cm in diameter. The mass, measuring 4 × 3.5 × 3.5 cm, was attached to an intact serosal surface. Microscopically, sections revealed an encapsulated lesion composed of bland-looking spindle and epithelioid cells with vesicular, round to ovoid nuclei (Fig. 3A and B). Inflammatory cells with mitotic figures were noted (0–1/HPF). Immunohistochemistry demonstrated strongly positive CD117 and DOG-1 immunostaining reactions (Fig. 3C and D). The second histopathology from the second look revealed an active inflammation, congestion, and reactive changes with congested mesenteric fat. The histopathology was negative for malignancy with a single negative lymph node.

The patient was discharged on postoperative day 19 and was given a referral to the national cancer centre for further assessment and management.

## 3 Discussion

GISTs are considered as rare tumours of the GI tract, accounting for approximately less than 0.1–3 % of GI neoplasms [1]. They arise from the interstitial cells of Cajal (ICC). ICC serve as pacemaker cells that control the motility of the GI tract [9]. Most of these tumours are benign, with only 30 % showing malignant potential [14]. Prior to the discovery of GISTs by Mazor and Clark in 1983, GISTs were formerly known as Gastrointestinal leiomyosarcomas (GI LMS). It was mistakenly identified as leiomyoma and leiomyosarcoma prior to the discovery of KIT testing [8], [9]. In terms of the location of the tumours, they can arise anywhere through the GI system, most commonly in the stomach (60 %), followed by the small bowel (20–30 %) and less than 10 % in the oesophagus, colon and rectum [2], [3]. Extra-GIST has also been reported in pancreas, gallbladder, liver and urinary bladder [10]. The possible root cause of GIST is the genetic mutation in one of several genes with almost 95 % of cases being linked to the mutation in the KIT gene, whereas only 3–5 % were associated with mutations in the PDGFRα gene [8]. The KIT gene, also known as c-kit gene encodes protooncogene c kit or CD117. CD117 is type III tyrosine kinase receptor that gets activated on the surface of different types of cells including hematopoietic stem cells, binding specifically to stem cell factor. Therefore, the overexpression of the KIT proto-oncogene leads to neoplasia [13]. The mutation of the KIT proto-oncogene tends to cluster in four exons (9,11,13,17), and the mutation of PDGRA genes mostly occurs in exons (12, 14, 18) [8]. Moreover, Discovered on GIST-1 (DOG-1) protein was identified as a highly sensitive and specific marker showing promising results in identifying GISTs, especially in CD117-negative tumours. It is a calcium-regulated chloride channel protein that is expressed on GIST cell membrane [14].

The spectrum of clinical presentation in GIST is broad, it is mainly dependent on the size of the tumour and site. Small-sized GISTs are usually asymptomatic which makes early diagnosis challenging. These are usually found incidentally on CT, endoscopy or intraoperatively. The symptoms present in such cases are non-specific and they include GI bleeding, anaemia, weight loss, vomiting, epigastric pain, as well as a palpable mass [1], [11]. Rarely, a small percentage of GISTs present acutely with GI bleeding, small bowel obstruction or perforation that require immediate surgical intervention [12]. Our case demonstrates a rare presentation of GIST in the form of a life-threatening GI bleeding. Therefore, we would like to emphasise the importance of recognising GIST as a differential in cases of acute GI bleeding where it could otherwise be misdiagnosed. Failure in early detection could lead to delay in life-saving treatment, resulting in increased risk of morbidity and mortality.

GISTs are usually discovered incidentally during radiologic or endoscopic investigations for an abdominal pathology or even during an abdominal surgery. This could be owed to the non-specific GI symptoms mentioned above. In our case, a perforated viscus was initially suspected given the patient's history of peptic ulcer disease along with the presenting symptoms. However, this had completely changed after conducting CTA, which created the impression of GIST. CT scan of the abdomen and pelvis is considered the imaging modality of choice for diagnosing and staging the primary tumour. These tumours appear as well-circumscribed, highly vascular mass with extraluminal growth pattern [15]. However, large tumours appear heterogenous, hypovascular, and can show areas of necrosis [16]. A definitive diagnosis of GIST is made by demonstrating the histopathological features and disease-related mutations. Histologically, three subtypes of GIST have been identified, including spindle cell (70 %), epithelioid (20 %), and mixed configurations, which were demonstrated in our case [15]. Most GISTs are strongly positive for CD-117 (KIT), which is crucial for the diagnosis of these tumours. Other immunohistochemical markers include CD34, DOG-1, SMA, S100, and Desmin [17].

Complete surgical resection with safety margins of 1-2 cm is considered the definitive therapy for localised GISTs [6]. Regional lymph node dissection is not indicated unless enlarged nodes are present since GIST rarely metastasizes to the lymph nodes. Mohamed et al. published a similar case of jejunal GIST (4x3x2 cm), diagnosed in a 58 year-old female, who had presented with hemodynamic instability and had been managed by surgical resection of the tumour [1]. The postoperative period was uneventful, and the patient was discharged in a stable state. Shi et al. on the other hand, reported a case of jejunal GIST (2 × 2.5 cm) in a 62 year-old male, who had presented with massive gastrointestinal bleeding and continuous drop in blood pressure [18]. The patient had been managed initially with urgent endoscopic sclerotherapy, 7 days after which surgery had been performed for complete resection of the tumour. In cases of GI bleeding caused by GIST, transcatheter arterial embolisation (TAE) can be effective in achieving hemostasis till definitive management with surgery is performed. The safety of this procedure was demonstrated in a study conducted in 20 cases of GIST, in whom TAE was performed and showed 90–95 % success rates [19]. However, such a procedure was not quite feasible in our case as the patient was hemodynamically unstable, necessitating an emergent surgical exploration.

Metastatic, recurrent, or unresectable tumours can be well managed with imatinib mesylate, a protein-tyrosine kinase inhibitor which has been successfully found to improve the overall survival and progression free survival as an adjuvant therapy [20]. In such cases, surgery can also be considered for the management of bleeding or obstruction and after a good response to imatinib therapy for locally advanced or unresectable tumours. Moreover, imatinib can be used as an adjuvant therapy for resectable tumours in patients with high risk of recurrence after complete tumour resection with no preoperative imatinib. Mutational testing should be conducted prior to the use of imatinib since GISTs respond differently based on different genotypes. For instance, mutations in exon 11 of the KIT gene confer a better response to imatinib when compared to exon 9 mutations at 400 mg dose. On the other hand, most PDGFRA mutations are responsive to imatinib, except for the D842V mutation, which only shows a response to avapritinib [21].

The prognosis of GIST is highly determined by the size of tumour and mitotic activity. Tumours larger than 5–10 cm or those with a mitotic activity of more than 10/50 HPF are associated with higher risk of malignancy and recurrence rate [20].

## 4 Conclusion

GISTs account for a minority of GI tumours; nonetheless, its recognition as a potentially life-threatening vascular tumour is crucial. It may often be misdiagnosed due to its rarity especially in cases of acute GI bleeding, which can result in a delay in life-saving treatment. CTA is the imaging modality of choice for visualisation of GIST and a definitive diagnosis is often dependent on histopathology. The management of GIST has certainly developed over the past years with the addition of chemotherapy as an adjuvant or neoadjuvant therapy to the mainstay surgical intervention. Finally, determining the prognosis is quite intricate, often dependent on factors such as the size and the mitotic activity of the tumour.

## Consent

Written informed consent was obtained from the patient for publication of this case report and accompanying images. A copy of the written consent is available for review by the Editor-in-Chief of this journal on request.

## Provenance and peer review

Not commissioned, externally peer-reviewed.

## Ethical approval

Not applicable.

## Funding

No funding or grant support for my case report.

## Author contribution

Nasrin Saeidi: literature review, writing, editing, manuscript drafting.

Yousef AlAli: literature review, writing, editing.

Reem Boushehry: literature review, writing, editing.

Sarah Al Safi: performed the surgery, critical review, supervision, final approval.

## Guarantor

Sarah Al Safi.

## Research registration number

Not applicable.

## Declaration of competing interest

There are no conflicts of interest including any financial or personal relationships with other people or organisations or any work influencers.

## References

[1] Mohamed A.A., Al Zahrani S.M., Mohamed S.A., Qureshi A.S. (2021). Massive gastrointestinal haemorrhage unusual presentation of gastrointestinal stromal tumors of the jejunum: case report and literature review. Cureus.

[2] Gerrish S.T., Smith J.W. (2008). Gastrointestinal stromal tumors-diagnosis and management: a brief review. Ochsner J..

[3] Miettinen M., Lasota J. (2003). Gastrointestinal stromal tumors (GISTs): definition, occurrence, pathology, differential diagnosis and molecular genetics. Pol.J.Pathol..

[4] Kermansaravi M., Rokhgireh S., Darabi S., Pazouki A. (2017). Laparoscopic total gastrectomy for a giant gastrointestinal stromal tumor (GIST) with acute massive gastrointestinal bleeding: a case report. Wideochir Inne Tech Maloinwazyjne.

[5] Feig Barry (2017).

[6] Manxhuka-Kerliu S., Sahatciu-Meka V., Kerliu I., Juniku-Shkololli A., Kerliu L., Kastrati M., Kotorri V. (2014). Small intestinal gastrointestinal stromal tumor in a young adult woman: a case report and review of the literature. J.Med..

[7] Agha R.A., Franchi T., Sohrabi C., Mathew G., for the SCARE Group (2020). The SCARE 2020 guideline: updating consensus Surgical CAse REport (SCARE) guidelines. Int. J. Surg..

[8] Tan C.B., Zhi W., Shahzad G., Mustacchia P. (2012). Gastrointestinal stromal tumors: a review of case reports, diagnosis, treatment, and future directions. ISRN Gastroenterol..

[9] Roy S.D., Khan D., De KK De U. (2012 Nov 29). Spontaneous perforation of jejunal gastrintestinal stromal tumour (gist). Case report and review of literature. World J. Emerg. Surg..

[10] Pandit N., Das G.P., Dahal M., Awale L. (2018 Dec 21). An unexpected extra-gastrointestinal stromal tumor (E-GIST) on the jejunal mesentery. J. Surg. Case Rep..

[11] Scherübl H., Faiss S., Knoefel W.T., Wardelmann E. (2014 Jul 16). Management of early asymptomatic gastrointestinal stromal tumors of the stomach. World J. Gastrointest. Endosc..

[12] Scola D., Bahoura L., Copelan A., Shirkhoda A., Sokhandon F. (2017 May). Getting the GIST: a pictorial review of the various patterns of presentation of gastrointestinal stromal tumors on imaging. Abdom. Radiol..

[13] Huret J.L., Ahmad M., Arsaban M., Bernheim A., Cigna J., Desangles F., Guignard J.C., Jacquemot-Perbal M.C., Labarussias M., Leberre V., Malo A. (2012 Nov 17). Atlas of genetics and cytogenetics in oncology and haematology in 2013. Nucleic Acids Res..

[14] Rammohan A., Sathyanesan J., Rajendran K., Pitchaimuthu A., Perumal S.K., Srinivasan U.P., Ramasamy R., Palaniappan R., Govindan M. (2013). A gist of gastrointestinal stromal tumors: a review. World J.Gastrointest.Oncol..

[15] van der Zwan S.M., DeMatteo R.P. (2005). Gastrointestinal stromal tumor: 5 years later. Cancer.

[16] Ashoor A.A., Barefah G. (2020 Feb 6). Unusual presentation of a large GIST in an extraintestinal site: a challenging diagnosis dilemma. BMJ Case Rep..

[17] Khuri Safi, Gilshtein Hayim, Darawshy Abd-alkarim, Bahouth Hany, Kluger Yoram (2017). Primary small bowel GIST presenting as a life-threatening emergency: a report of two cases. Case Rep. Surg..

[18] Shi Xiuju, Yu Shuxia, Wang Fenyan, Zhao Qi, Xu Hongwei, Li Bin (March 2018). A gastrointestinal stromal tumor with acute bleeding: management and nursing. Medicine.

[19] Koo H.J., Shin J.H., Shin S., Yoon H.K., Ko G.Y., Gwon D.I. (2015 Sep). Efficacy and clinical outcomes of transcatheter arterial embolization for gastrointestinal bleeding from gastrointestinal stromal tumor. J. Vasc. Interv. Radiol..

[20] Sashidharan P., Matele A., Matele U., Al Felahi N., Kassem K.F. (2014 Mar). Gastrointestinal stromal tumors: a case report. Oman Med. J..

[21] National Comprehensive Cancer Network (NCCN) NCCN Clinical Practice Guidelines in Oncology. Gastrointestinal Stromal Tumors (GISTs) Version 1.2022 — January 21, 2022; NCCN Guidelines for Patients. http://www.nccn.org/patients.

